# Pleurésie purulente à Nocardia asteroids

**DOI:** 10.11604/pamj.2014.18.346.5132

**Published:** 2014-08-28

**Authors:** Régis Gothard Bopaka, Hind Janah, Wiam El Khattabi, Abdelaziz Aichane, Hicham Afif

**Affiliations:** 1 Service des Maladies Respiratoires, Hôpital « 20 août 1953 », CHU Ibn Rochd, Casablanca, Maroc

**Keywords:** Pleurésie purulente, Nocardia asteroides, diabète, Empyema, Nocardia asteroides, diabetes

## Abstract

La pleurésie purulente à *Nocardia asteroides* est rare. Elle survient le plus souvent sur un terrain d'immunodépression. Nous rapportons une observation d'une pleurésie à *Nocardia asteroides* chez une patiente âgée de 60 ans, chez qui nous avons découvert un diabète. A travers ce travail les auteurs soulignent l'intérêt de la multiplication de prélèvements, de rechercher ce germe sur terrain d'immunodépression et en cas d'isolement, de rechercher la comorbidité.

## Introduction

La pleurésie purulente est due à des germes pyogènes dont les plus fréquents sont les streptococcus pneumonia et les staphylococcus aureus. Cependant certains germes sont rares tels que le *Nocardia asteroides*. La pleurésie purulente à*Nocardia astéroides* est rare [1]. Nous rapportons une observation médicale d'une patiente présentant une pleurésie purulente à *Nocardia asteroides*.

## Patient et observation

Il s'agit d'une patiente âgée de 60 ans, femme au foyer. Elle était sans antécédents pathologiques particuliers, notamment non connue porteuse suivie pour diabète, et n'ayant pas d'autres comorbidité, non suivie pour une néoplasie. Elle présentait une pleurésie purulente récidivante évoluant depuis trois mois. Elle a été traitée par une antibiothérapie à base d'amoxicilline associée à l'acide clavulanique 3g/j pendant 8 semaines puis par bi antibiothérapie associant le métronidazole 1,5g/j pendant 10 jours sans succès.

L'examen clinique à l'admission, la patiente était apyrétique, asthénique, polypnéique à 28 cycles/min et tachycarde à 110 batt/min. L'examen pleuro-pulmonaire a objectivé un syndrome d’épanchement liquidien basithoracique droit. L'auscultation cardio-vasculaire était normale. L'examen de l'abdomen était normal et l'examen buccodentaire était normal. Le reste de l'examen somatique était sans particularité.

**Figure 1:**
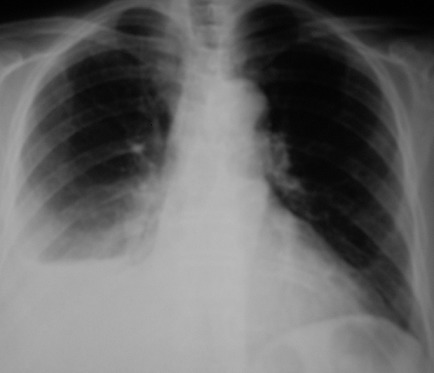
Pleurésie droite à l'admission

La radiographie thoracique a montré une opacité de type pleurale basale droite (Figure 1) moins typique d'autant que sa limite supérieure est concave en haute mais rectiligne en dedans. L’échographie thoracique a montré l’épanchement du type pleural. La ponction pleurale exploratrice a mis en évidence un liquide purulent. Des prélèvements bactériologiques étaient réalisés. La numération formule sanguine a montré une hyperleucocytose à 15660/mm3 à prédominance polynucléaire neutrophile (12085/mm3) sans lymphocytaire. La vitesse de sédimentation était accélérée à 70mm. L'analyse du liquide pleural a montré un liquide d'aspect purulent avec 80% de polynucléaires neutrophiles altérés. La recherche de bacille de Koch à l'examen direct et à la culture dans le liquide pleurale était négative. L'intradermoréaction à la tuberculine était négative. Le bilan rénal était normal. Le bilan hépatique était normal. Les sérologies virales hépatiques B, C et de l'immunodéficience humain (VIH) étaient négatives. La glycémie à jeun était à 4,35 g/l. La bandelette urinaire a montré la présence de glucose, de protéine et absence de corps cétoniques. Le dosage de l'hémoglobine glyquée était à 7%.

La patiente était mise sous tri-antibiothérapie probabiliste à base d'amoxicilline-acide clavulanique à 3g/j, de moxifloxacine à 400 mg/j et de métronidazole à 1,5 g/j. La ponction pleurale évacuatrice biquotidienne était instaurée ainsi que la kinésithérapie respiratoire. L'urgence hyper-glycémique était de démarrer l'insulinothérapie était administrée en concertation avec les endocrinologues face au diabète type 2. Devant la non amélioration radioclinique et la persistance et/ou récidive de l’épanchement, nous avons multiplié des prélèvements pleuraux à visées bactériologiques. L’échographie abdomino-pelvienne était normale sans autres anomalies visibles. Le résultat du 4ème prélèvement du liquide pleural a montré à l'examen direct la présence de bacilles à filaments. La culture a isolé le germe *Nocardia asteroides*. Le germe était sensible à la Gentamicine, Ciprofloxacine, Ceftriaxone, Triméthoprime Sulfamides.

**Figure 2:**
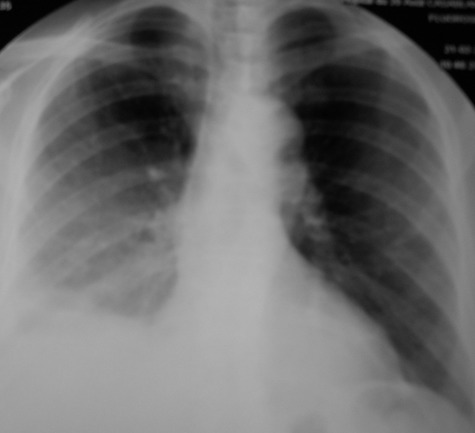
Pleurésie droite à 12 jours du traitement

**Figure 3:**
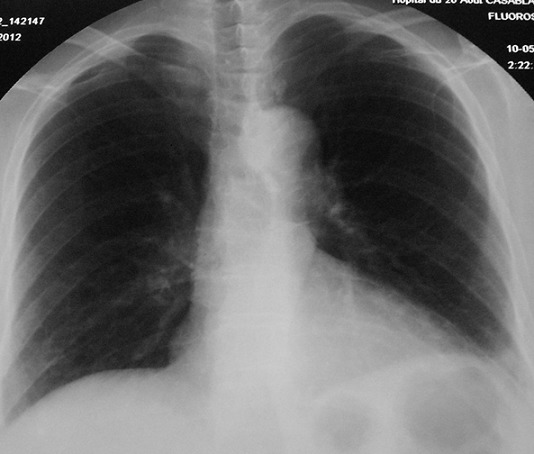
Évolution à trois mois du traitement

Le diagnostic de pleurésie purulente à *Nocardia asteroides* sur diabète type II était retenu. Le traitement était ajusté par l'introduction de cotrimoxazole (800mg/j) avec arrêt des antibiotiques antérieurs. L’évolution au 12ème jour était favorable au plan clinique par la disparition de la douleur thoracique droite, un bon état général, une régression du syndrome d’épanchement liquidien. La ponction pleurale évacuatrice a ramené au totale 1700ml. L’évolution biologique était bonne (Tableau 1). Une amélioration moins nette des images radiologiques (Figure 2). A trois mois du traitement de cotrimoxazole (800mg/j), l’évolution radiologique était, marquée par une disparition de la pleurésie (Figure 3). Le traitement de cotrimoxazole était arrêté.

**Tableau 1:** Évolution biologique

	1^er^ jour	15^ème^ jour
Globules blancs (/mm^3^)	15660	9200
Polynucléaire neutrophile (/mm^3^)	12085	6450
VS (mm à la 1^ere^ heure)	70	30
Glycémie (g/l)	4,35	1,45

## Discussion

La *Nocardia asteroides* est une bactérie gram positive, aérobie stricte [2]. Il atteint l'appareil respiratoire, l'appareil digestif, la peau, et le cerveau. Son incidence annuelle aux Etats-Unis est évaluée à environ 1/250 000 habitants [1]. La nocardiose est le plus souvent sous-estimée, et sous diagnostiqué. En cas d'atteinte de l'appareil respiratoire, il s'agit de nocardiose pulmonaire. L'atteinte pleurale est rare. Les signes cliniques sont non spécifiques, souvent assimilés à la tuberculose. De même il s'agit d'une bactérie acido-alcolo-résistante comme le bacille de Koch. Le syndrome d’épanchement liquidien peut être retrouvé tant dans la pleurésie tuberculeuse que dans la pleurésie à *Nocardia asteroides* avec tous un début progressif. La *Nocardia asteroides* est un germe ubiquitaire. Les atteintes des autres appareils doivent être systématiquement recherchés: atteinte digestive (hépato-splénomégalie); atteinte cutanée (nodule cutané, abcès cutané); atteinte neurologique (céphalées, confusion, coma). Aucun de ces signes n’était retrouvé chez notre patiente.

Devant toute pleurésie purulente, la recherche de facteurs de risques s'impose et cela est de règle pour la pleurésie purulente à *Nocardia asteroides*. Les facteurs de risque exposent les patients à de germes opportunistes. La nocardiose se voit principalement chez des sujets immunodéprimés, les patients sous corticoïdes, le VIH/SIDA, une néoplasie, les pathologies respiratoires (Broncho-pneumopathie chronique obstructive), la tuberculose et le diabète [3–6]. Notre patiente était diabétique, de découverte récente au cours de son hospitalisation. La nocardiose est un germe saprophyte, pathogène [1], des voies aériennes supérieures qui se disséminent en cas d'immunodépression. L'atteinte pleurale fait suite à l'ensemencement de la cavité pleurale, le plus souvent par contiguïté à une atteinte pulmonaire par voie lymphatique [1]. Tel est le cas probablement dans notre observation. L'atteinte respiratoire par voie digestive suite à l'ingestion d'aliment contaminé ou mal lavé (le *Nocardia asteroides* est un germe tellurique) est rare [2].

Le diagnostic repose sur la confirmation bactériologique par l'isolement du germe à bacille gram positif, à filaments ramifiés. La culture en milieu anaérobie des prélèvements permet d'isolé le germe. Le prélèvement du liquide pleural a permis dans notre cas d'isolement le germe, car d'autres sites de prélèvements sont possibles comme les prélèvements bronchiques, du liquide céphalorachidien.

Le traitement peut être médical ou chirurgical. Le traitement médical a été envisagé chez notre patiente. Et il repose sur l'antibiothérapie à base de sulfamide, ou de ciprofloxacine, sans oublier l’évacuation de l’épanchement et de la kinésithérapie respiratoire. La patiente a reçu les antibiotiques probabilistes ensuite adapter en fonction de l'antibiogramme [7] notamment du cotrimoxazole. Le cotrimoxazole est un traitement de référence [8, 9]. Le germe était résistant à l'amoxicilline, comme le plus décrit dans la littérature. La durée du traitement était de 3 mois chez notre patiente. Cette durée du traitement est variable et il n'y a pas de consensus. Une durée est souhaitable car un traitement trop court est parfois à l'origine des récidives [7]. C'est une raison où notre patiente avait une pleurésie purulente récidivante avant son admission.

## Conclusion

La pleurésie purulente à *Nocardia astéroides* est une infection rare. Le plus souvent sous-diagnostiqué. Elle survient le plus souvent sur un terrain d'immunodépression. A travers cette observation, nous soulignons l'intérêt de la multiplication des prélèvements et la recherche systématique de facteurs favorisants devant toute pleurésie purulente persistante.
